# Picornavirus security proteins promote the release of extracellular vesicle enclosed viruses via the modulation of host kinases

**DOI:** 10.1371/journal.ppat.1012133

**Published:** 2024-04-25

**Authors:** Kyra A. Y. Defourny, Xinyi Pei, Frank J. M. van Kuppeveld, Esther N. M. Nolte-´t Hoen

**Affiliations:** 1 Infection Biology Section, Division of Infectious Diseases & Immunology, Department of Biomolecular Health Sciences, Faculty of Veterinary Medicine, Utrecht University, Utrecht, The Netherlands; 2 Virology Section, Division of Infectious Diseases & Immunology, Department of Biomolecular Health Sciences, Faculty of Veterinary Medicine, Utrecht University, Utrecht, The Netherlands; University of North Carolina at Chapel Hill School of Medicine, UNITED STATES

## Abstract

The discovery that extracellular vesicles (EVs) serve as carriers of virus particles calls for a reevaluation of the release strategies of non-enveloped viruses. Little is currently known about the molecular mechanisms that determine the release and composition of EVs produced by virus-infected cells, as well as conservation of these mechanisms among viruses. We previously described an important role for the Leader protein of the picornavirus encephalomyocarditis virus (EMCV) in the induction of virus-carrying EV subsets with distinct molecular and physical properties. EMCV L acts as a ‘viral security protein’ by suppressing host antiviral stress and type-I interferon (IFN) responses. Here, we tested the ability of functionally related picornavirus proteins of Theilers murine encephalitis virus (TMEV L), Saffold virus (SAFV L), and coxsackievirus B3 (CVB3 2A^pro^), to rescue EV and EV-enclosed virus release when introduced in Leader-deficient EMCV. We show that all viral security proteins tested were able to promote virus packaging in EVs, but that only the expression of EMCV L and CVB3 2A^pro^ increased overall EV production. We provide evidence that one of the main antiviral pathways counteracted by this class of picornaviral proteins, i.e. the inhibition of PKR-mediated stress responses, affected EV and EV-enclosed virus release during infection. Moreover, we show that the enhanced capacity of the viral proteins EMCV L and CVB3 2A^pro^ to promote EV-enclosed virus release is linked to their ability to simultaneously promote the activation of the stress kinase P38 MAPK. Taken together, we demonstrate that cellular stress pathways involving the kinases PKR and P38 are modulated by the activity of non-structural viral proteins to increase the release EV-enclosed viruses during picornavirus infections. These data shed new light on the molecular regulation of EV production in response to virus infection.

## Author summary

During infection, virus particles can be packaged by host cells within extracellular vesicles (EVs), nanosized vesicles that serve as vehicles for cell-to-cell communication. These EVs can shield virus particles from antibodies, and alter the uptake of viruses by susceptible cells and cells carrying-out immune surveillance. However, despite the potential consequences of EV-enclosed virus release on disease progression, little is known about the virus and host factors regulating this process. Here, we investigated the role of virus-encoded host-modulating proteins in the packaging of non-enveloped viruses in EVs, focusing on the *picornavirus* virus family. We previously demonstrated that the picornavirus encephalomyocarditis virus alters the number and type of EVs released by infected cells to promote EV-enclosed virus release, and that this depended on the activity of the viral Leader protein. Using a panel of knockout cells, inhibitors, and recombinant virus constructs, we here show that this alteration of EV release depends on modulation of the pathways downstream of two host stress kinases, PKR and P38 MAPK, and is conserved in the 2A protease of distantly related pathogen coxsackievirus B3. These data shed light on the molecular regulation of EV-enclosed virus release, and pave the way to find inhibitors of EV-mediated virus spread.

## Introduction

A growing number of non-enveloped viruses are known to be released from infected cells in two forms: as naked virions, and as quasi-enveloped virus particles enclosed in host-derived membranes. In contrast to enveloped viruses, non-enveloped viruses lack specialized transmembrane proteins to induce membrane budding and release. Instead, these viruses make use of the host’s natural ability to produce membrane vesicles, also known as extracellular vesicles or EVs, to induce the release of virions in a membrane-enclosed form. EVs are naturally released by all cells as a means of cell-to-cell communication [1]. Whereas the release of naked virions typically requires lysis of the host cell, the packaging of virions within EVs allows viruses to escape the host cell prior to the rupture of cellular membranes [2–5]. This process results in the formation of infectious virus particles that differ in functional properties compared to their naked counterparts. Depending on the exact composition of the EV carrier, including the number of enclosed virus particles, EV-enclosed viruses can display altered uptake and infection dynamics [6–12]. Importantly, EV-enclosed virus particles become inaccessible for neutralizing antibodies [13–17]. Hence, EVs constitute long overlooked players in the viral life cycle as well as a determinant of infection progression.

Initial studies have provided first insight into how the release of EV-enclosed virus is regulated. Although virions may be packaged in EVs that are constitutively released by cells, many viruses were found to alter EV release by the host cell. The non-enveloped viruses encephalomyocarditis virus (EMCV), rotavirus, astrovirus, enterovirus A71, and hepatitis A virus (HAV) were shown to increase EV production during infection [4,18–21]. Moreover, we previously demonstrated that EMCV infection induced the release of specific EV subsets with distinct protein composition, physical properties, and potential to infect cells [4]. For HAV, EV-enclosed virus release was shown to be promoted by an interaction between the EV biogenesis protein Alix and a motif within the viral capsid [20]. In contrast, for other members of the family *Picornaviridae*, most notably EMCV, poliovirus, and coxsackievirus B3 (CVB3), the autophagy machinery has been implicated in the packaging of virions in EVs [2,22–25]. These viruses trigger an unconventional arm of the autophagy pathway, termed secretory autophagy, via which autophagosomal cargo is delivered to the extracellular environment instead of the lysosomal degradation pathway [26]. We previously demonstrated that the ability of EMCV to induce secretory autophagy as well as the release of EVs and EV-enclosed virus required the expression of the non-structural viral Leader (L) protein [25]. However, the mechanisms via which EMCV L, and possibly also non-structural proteins of other picornaviruses, modulate host cells to enable the release of virus-containing EVs are unknown.

EMCV L is a multifunctional protein that is dispensable for virus replication but is key to altering host cell signaling and function during infection [27]. The activity of EMCV L is crucial for the suppression of type-I interferon induction, which is primarily mediated by the disruption of the nucleopore complex resulting in nucleocytoplasmic trafficking disorder (NCTD) [28]. In addition, EMCV L suppresses the integrated cellular stress response and formation of stress granules during infection [29,30]. To mediate these effects, EMCV L directly or indirectly modulates the activity of host cell kinases [31–34]. Notably, non-structural proteins of many other picornaviruses have evolved to target similar host processes via diverse molecular mechanisms, despite sharing little to no overlap in sequence or structure. These evolutionary heterogenous picornaviral proteins have previously been referred to as “viral security proteins”, based on their shared functions in counteracting host-defense mechanisms [35]. The security proteins that share most overlap with EMCV L (±40% amino acid similarity), are the L proteins of Theiler’s murine encephalitis virus (TMEV) and Saffold virus (SAFV), which together with EMCV form the genus *Cardiovirus* within the *Picornaviridae* [36]. The cardiovirus L proteins were previously reported to be functionally interchangeable, as EMCV L was able to modulate host defense and induce NCTD when replacing TMEV L in the TMEV genome [29,37,38]. The same functional interchangeability has been demonstrated for more distant picornavirus security proteins, as the 2A protease (2A^pro^) of the enterovirus CVB3 was able to suppress stress granule formation and IFN induction when exchanged with EMCV L, despite the lack of similarity in sequence or structure of these proteins [38].

In this study, we first investigated whether different picornavirus non-structural proteins that are known to subvert the same cellular defense mechanisms also share the ability to promote the release of EV-enclosed viruses. In our comparison we included EMCV L, TMEV L, SAFV L, and CVB3 2A^pro^, which all suppress SG formation and type I IFN production while inducing NCTD. The ability of TMEV L, SAFV L, and CVB3 2A^pro^ to promote EV-enclosed virus release was tested using a recombinant virus system in which we inserted these viral proteins into an EMCV strain lacking L protein activity. We show that all tested proteins could promote the release of EV-enclosed EMCV, but with different efficiencies. Only EMCV L and CVB3 2A^pro^ could trigger alterations in the number and composition of EVs released by infected cells and induced the release of a distinct virus-carrying EV subset. We subsequently made use of the functional commonalities and differences between these viral security proteins to identify host processes and signaling pathways that affect the release of EV-enclosed viruses. Using knockout and chemical inhibition strategies, we demonstrate a role for PKR and P38 MAPK signaling pathways in determining the efficiency with which EV-enclosed viruses are released in response to virus infection.

## Results

### The activities of picornavirus security proteins EMCV L, TMEV L, SAFV L, and CVB3 2A^pro^ promote EV-enclosed virus release

**Fig 1:**
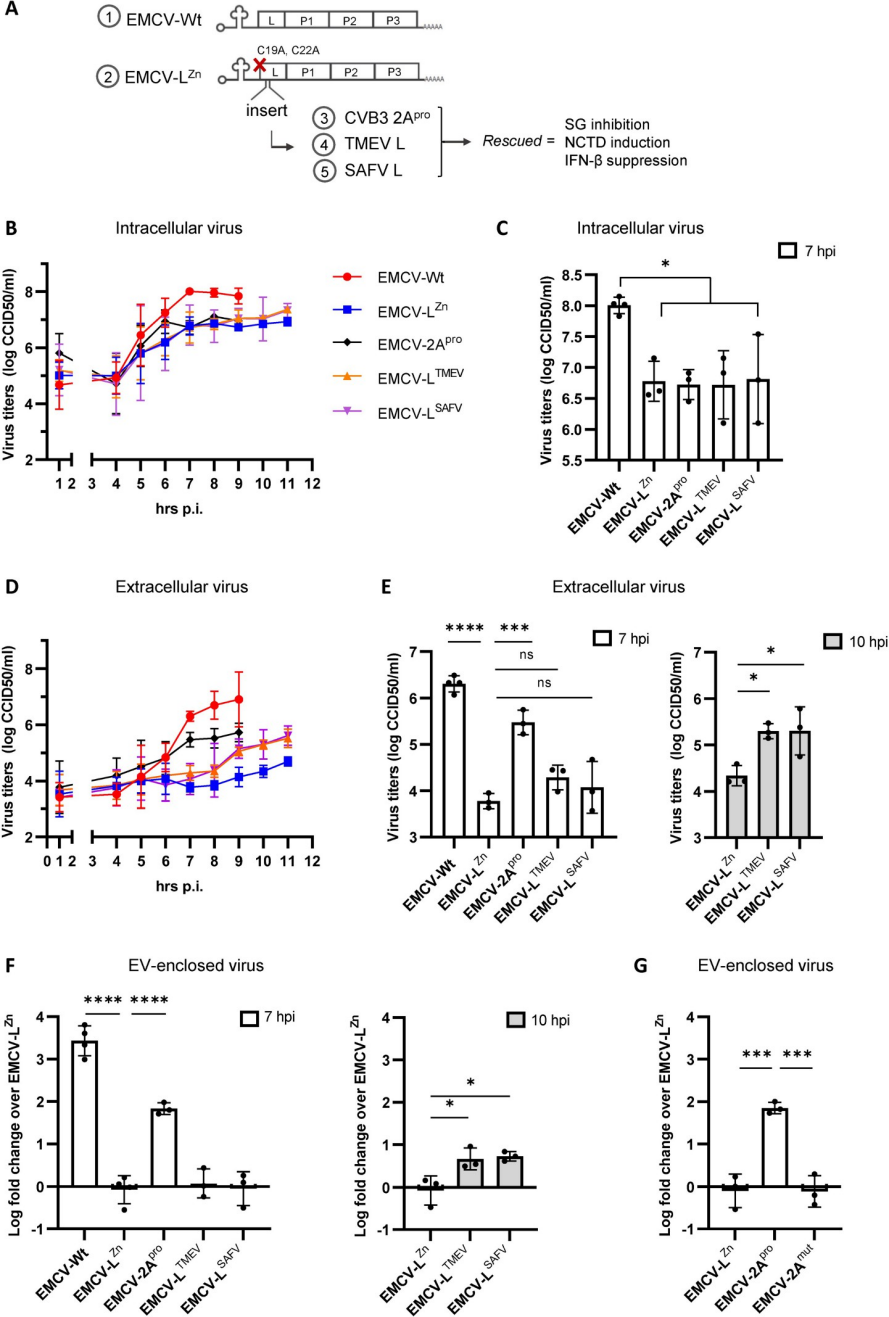
The picornavirus security proteins CVB3 2A^pro^, TMEV L, and SAFV L promote EV-enclosed virus release with different efficiencies. (A) Depicted is a schematic overview of the recombinant viruses used in this study. To generate these viruses, the coding sequences of TMEV L (L^TMEV^), SAFV L (L^SAFV^), or CVB3 2A (2A^pro^) proteins were fused to the polyprotein of an EMCV strain containing an inactivating mutation in the EMCV L protein (EMCV-L^Zn^), separated by a 3C cleavage site. (B-G) HeLa cells were infected with the viruses described in (A) at MOI 10. (B) Intracellular virus titers were determined over time by end-point dilution assay and are depicted as line graph. In (C) a bar graph is depicted comparing the intracellular virus titers at 7 hpi. Corresponding extracellular virus titers are depicted in (D) and (C), with bar graphs comparing the extracellular virus titers at 7 hpi (left) and 10 hpi (right). (F) EVs released by cells infected with EMCV-Wt, EMCV-L^Zn^, or EMCV-L^Zn^ reconstituted with the indicated viral proteins were isolated by density gradient centrifugation at 7 or 10 hpi. EV-enclosed virus titers were determined by end-point dilution assay. Data are expressed as fold increase in EV-enclosed virus titers relative to EMCV-L^Zn^. (G) EV-enclosed virus release at 7 hpi was compared for EMCV-L^Zn^ reconstituted with proteolytically active *vs*. proteolytically inactive CVB3 2A^pro^ (EMCV-2A^pro^ and EMCV-2A^mut^ respectively). Bar and line graphs depict means ± SD of n = 3–4 independent experiments. *p<0.05, ***p<0.0005, ****p<0.0001 as determined by one-way ANOVA with Tukey’s multiple comparisons test.

We previously showed that EMCV L plays an important role in the release of virus-carrying EV subsets during EMCV infection [25]. To investigate whether this function is shared among security proteins of related picornaviruses, we employed a recombinant virus system in which different viral security proteins could be expressed in the context of the EMCV genome. In this system, genes encoding the L proteins of the cardioviruses TMEV and SAFV, as well as the 2A^pro^ protein of the enterovirus CVB3, were inserted into the genome of mutant EMCV carrying an inactivating mutation in its own L protein (EMCV-L^Zn^) (Fig 1A). EMCV-L^Zn^ is unable to suppress PKR activation and downstream stress granule formation, and fails to induce NCTD to suppress IFN-β production, thereby allowing the activation of these two important antiviral pathways during infection [30,39]. As anticipated, we observed that insertion of TMEV L (EMCV-L^TMEV^), SAFV L (EMCV-L^SAFV^), and CVB3 2A^pro^ (EMCV-2A^pro^) could restore the capacity of EMCV-L^Zn^ to modulate these host defense processes (S1 Fig) [28,35]. Notably, whereas the three Leader proteins directly inhibit PKR activation, we confirmed that CVB3 2A^pro^ suppresses the PKR antiviral pathway downstream of PKR (S1 Fig). The replication kinetics of recombinant EMCV-L^Zn^ viruses expressing TMEV L, SAFV L or CVB3 2A^pro^ were similar to EMCV-L^Zn^ (Fig 1B), as no significant difference in virus yields could be observed at any of the time points tested. However, intracellular virus titers for EMCV-Wt were significantly higher than for the other viruses from 7 hpi onwards (Fig 1C). We used EMCV-Wt and these recombinant viruses to investigate whether TMEV L, SAFV L, and CVB3 2A^pro^ share with EMCV L the ability to promote EV-enclosed virus release, and to determine if known functions ascribed to these proteins are important for this process.

We first assessed whether expression of the different picornavirus security proteins influenced total extracellular virus release. Expression of functional EMCV L (EMCV-Wt) or CVB3 2A^pro^ (EMCV-2A^pro^) significantly increased EMCV virus release compared to that observed for EMCV-L^Zn^ starting at 7 hpi (Fig 1D and 1E). EMCV L and CVB3 2A^pro^ proteins were found to increase virus release with a similar efficiency when values were corrected for the respective differences in intracellular virus titers (S2 Fig). In contrast, expression of TMEV L and SAFV L increased virus release to a lesser extent and only from 9–10 hpi (Fig 1D and 1E). This increase in virus release preceded a strong increase in the permeability of the infected cells, which occurred at 8 hpi for EMCV-Wt and EMCV-2A^pro^ infected cells and at 11 hpi for EMCV-L^TMEV/SAFV^ infected cells (S3 Fig). To assess if the increase in overall virus release observed at these time points correlated with an increase in the release of EV-enclosed virus, EVs were isolated from the cell culture supernatant of infected cells and separated from naked virions using density gradient centrifugation as described previously [4]. EMCV-Wt and EMCV-2A^pro^ induced a significant increase in EV-enclosed virus release at 7 hpi compared to EMCV-L^Zn^ (Fig 1F). This effect was larger for EMCV-Wt, in correspondence with the higher intracellular virus titers in EMCV-Wt infected cells (Fig 1C). At 10 hpi also the incorporation of TMEV L and SAFV L in EMCV-L^Zn^ enhanced EV-enclosed virus release, although to a lesser extent than observed for CVB3 2A^pro^ at 7 hpi (Fig 1F). In contrast to the cardiovirus leaders, CVB3 2A^pro^ is a viral protease. Both protease-dependent and independent functions have been attributed to the enterovirus 2A protein [38,40–42]. Mutation of the catalytic domain of CVB3 2A^pro^ did not affect intracellular virus titers (S4A Fig), but abolished its positive effect on EV-enclosed virus release, indicating that the ability of CVB3 2A^pro^ to promote EV-enclosed virus release required its proteolytic activity (Fig 1G). Based on these data, we conclude that the viral security proteins TMEV L, SAFV L, and CVB3 2A^pro^ are all able to promote EV-enclosed virus release during EMCV infection. Notably, the impact of TMEV L and SAFV L on EV-enclosed virus release was delayed and less pronounced (4.6 and 5.4 mean fold increase at T = 10) compared to that of proteolytic active CVB3 2A^pro^ (67.6 mean fold increase at T = 7) or EMCV L (2691.5 mean fold increase at T = 7, with an accompanying 23.3 fold increase in intracellular virus).

### The activities of EMCV L and CVB3 2A^pro^, but not TMEV L or SAFV L, increase the release of distinct EV subsets during infection

**Fig 2:**
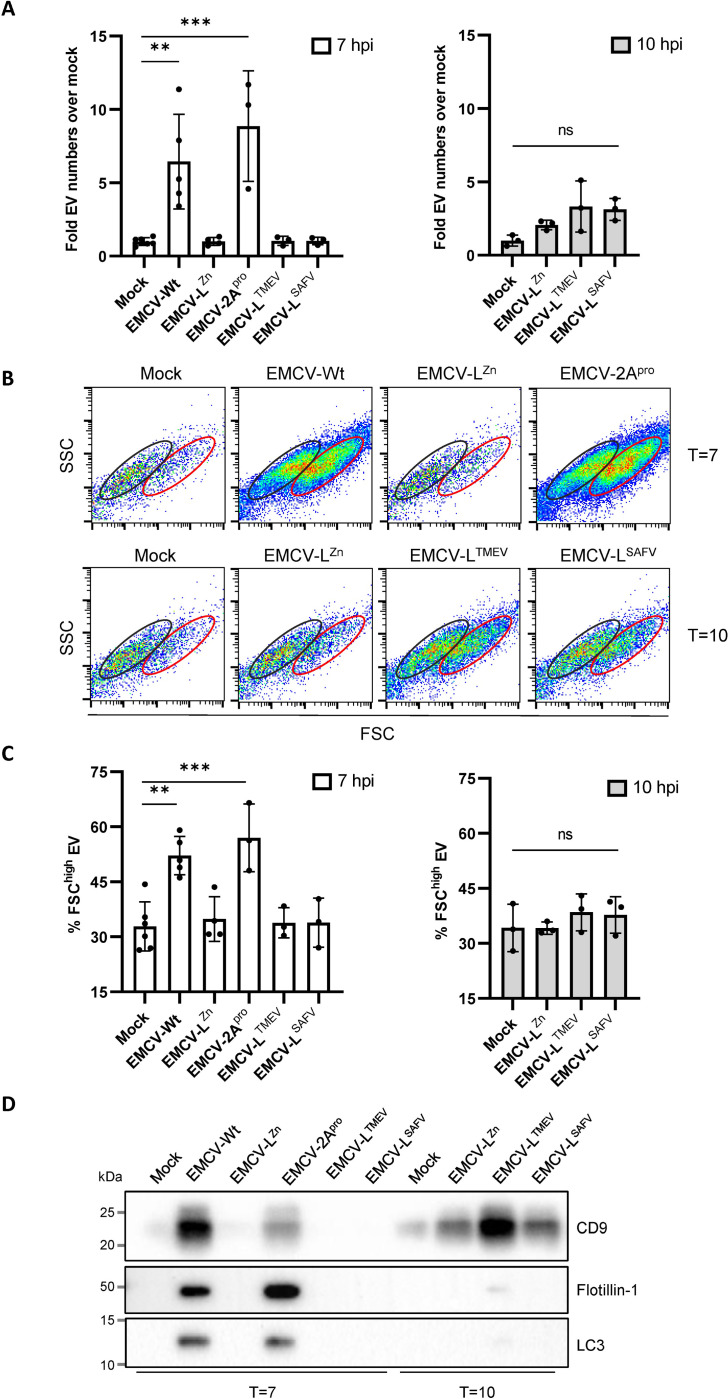
CVB3 2A^pro^ and EMCV L, but not TMEV L or SAFV L, strongly increase the release of virus-induced EV subsets carrying flotillin-1 and LC3. EVs were isolated by means of density gradient centrifugation at 7 or 10 hpi with EMCV-Wt, EMCV-L^Zn^, or the recombinant viruses EMCV-2A^pro^, EMCV-L^TMEV^, and EMCV-L^SAFV^. (A) EVs were fluorescently labeled with CFSE and quantified by high resolution flow cytometry. Depicted is the increase in EV numbers relative to the mock-infected control. Means ± SD of n = 3–4 independent experiments are shown. (B) Dot plots showing the forward (FSC) *vs*. side-scatter (SSC) profile of EVs in the peak fraction of samples corresponding to (A). (C) Bar graphs displaying the % of total EVs that fall within the FSC^high^ gate indicated in (B). (D) Western blot analysis of CD9, flotillin-1, and LC3 in density gradient purified EVs isolated from an equal number of cells. Depicted are representative images of n = 2 independent experiments. p**<0.005, ***p<0.0001 as determined by one-way ANOVA with Tukey’s multiple comparisons test.

Based on the differences observed in EV-enclosed virus release, we next set out to study the impact of the different viral security proteins on the number and type of EVs released by the infected cells. We applied high-resolution flow cytometry to analyze the quantity and quality of EVs released during infection at a single particle level [4,43,44]. Using this technique, we observed a 5- to 10-fold increase in the number of released EVs upon infection with EMCV-2A^pro^, which is similar to the induction of EV release observed during EMCV-Wt infection (Fig 2A). In line with our observations on EV-enclosed virus release (Fig 1E), the impact of CVB3 2A^pro^ on EV release was dependent on its protease activity (S4B Fig). However, expression of TMEV L or SAFV L did not restore induction of EV release by EMCV-L^Zn^ infected cells at 7 or 10 hpi (Fig 2A). In EV isolates from EMCV-Wt and EMCV-2A^pro^ infected cells, two EV populations were observed that induced different levels of forward scattered light (FSC) (Fig 2B). Our previous data indicate that FSC-high EVs contribute most to the spread of EMCV infection to new cells [4]. An increase in the percentage of FSC-high EVs was observed during infection with EMCV-Wt and EMCV-2A^pro^, but not with TMEV L or SAFV L expressing recombinant viruses (Fig 2C). This suggests that the activity of EMCV L and CVB3 2A^pro^, but not the other cardiovirus L proteins, can promote the release of distinct EV subsets in response to EMCV infection.

Western blot was performed to compare the protein composition of the EVs released by cells infected with the different viruses. Infection with EMCV-2A^pro^ enhanced the release of EVs carrying the tetraspanin CD9 and flotillin, resembling the EV subpopulation(s) induced by EMCV-Wt infection (Fig 2D). Moreover, LC3 could be detected in the EVs from cells infected with EMCV-Wt or EMCV-2A^pro^. More specifically, we detected a band corresponding to LC3-II, the lipidated form of LC3 (S5 Fig). LC3 is an autophagy-related protein, and its incorporation in EV marks the activation of a secretory arm of the autophagy pathway that we previously linked to virus packaging in EVs [25]. In contrast, EMCV-L^TMEV^ and EMCV-L^SAFV^ did not prominently enhance the release of flotillin and LC3 at 7 or 10 hpi (Fig 2D), although infection with EMCV-L^TMEV^ increased the release of CD9 at 10 hpi. These data indicate that along with the rapid induction of EV-enclosed virus release, CVB3 2A^pro^ shares with EMCV L the ability to efficiently promote the release of infection-related EV subsets, which are marked by a FSC-high phenotype and the presence of flotillin and LC3-II. The activity of TMEV L or SAFV L, on the other hand, only moderately promotes virus packaging in EVs at a later stage of the infection without an accompanying increase in overall EV release. Combined, these data indicate that security proteins from various picornaviruses promote the release of EV-enclosed EMCV, but with different efficiencies. Only EMCV L and CVB3 2A^pro^ induced alterations in the number and composition of EVs released by infected cells and induced the release of a distinct virus-carrying EV subset.

### Suppression of PKR activity, but not MAVS-mediated antiviral signaling, potentiates the release of EVs and EV-enclosed virus during EMCV infection

**Fig 3:**
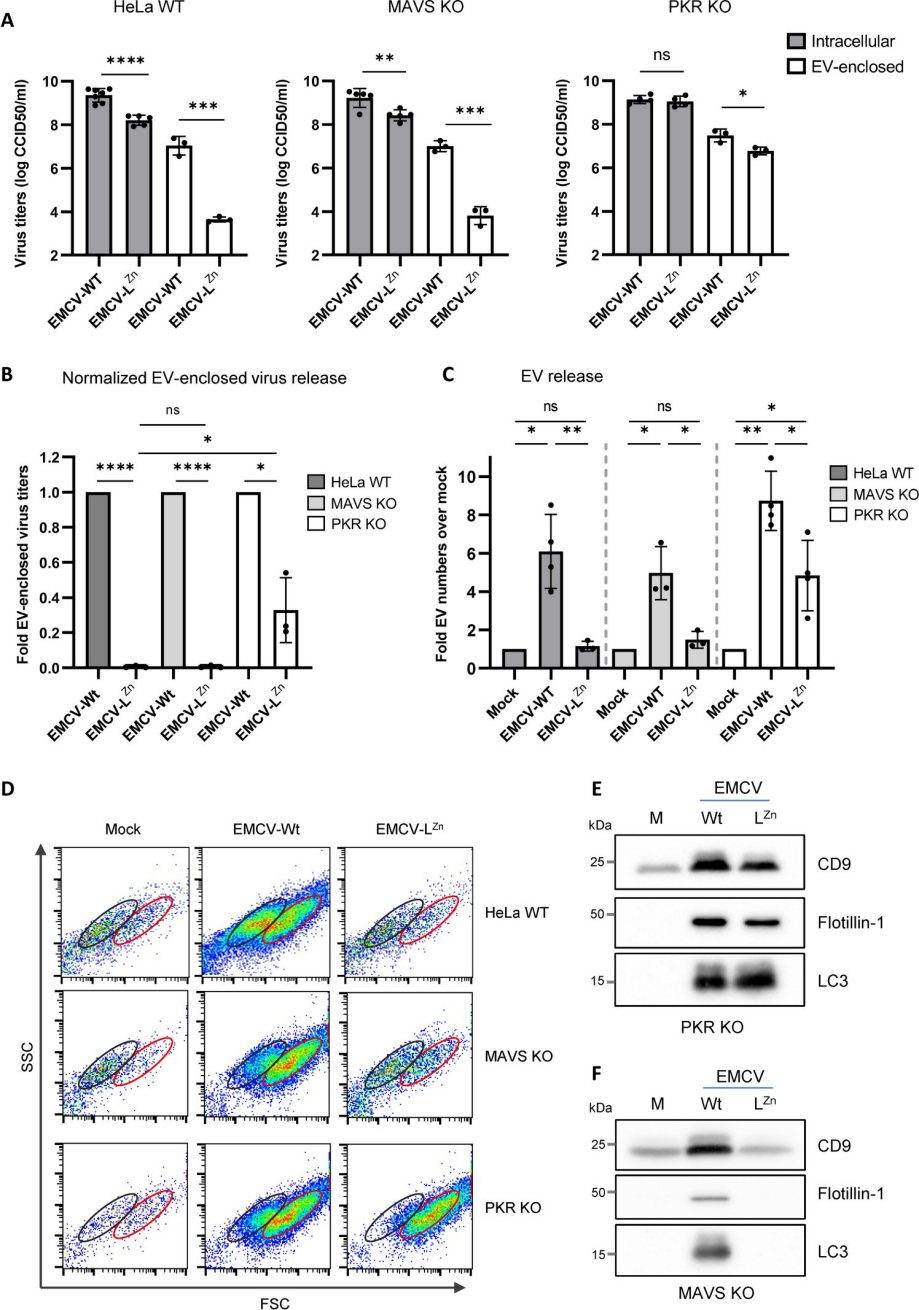
Suppression of PKR but not MAVS antiviral signaling potentiates the release of EVs and EV-enclosed virus during EMCV infection. WT, MAVS KO, and PKR KO cells were infected with EMCV-Wt or EMCV-L^Zn^. 7 hpi EVs were isolated by density gradient centrifugation to assess if inhibition of MAVS/PKR-mediated antiviral signaling cascades can rescue EV-enclosed virus release. (A) Intracellular and EV-enclosed virus titers were determined by end-point dilution assay. (B) Bar graphs displaying the fold difference in EV-enclosed virus titers detected in EMCV-L^Zn^ compared to EMCV-Wt infected samples, after correction for the corresponding differences in intracellular virus titers. Means ± SD of n≥3 independent experiments are shown. (C) EV numbers were quantified by high resolution flow cytometry. Depicted is the increase in EV numbers relative to the mock-infected controls. Means ± SD of n = 4 independent experiments are shown. (D) Dot plots showing the forward (FSC) *vs*. side-scatter (SSC) profile of EVs in the peak fraction of samples corresponding to (C). (E,F) EVs isolated by ultracentrifugation from an equal number of (mock) infected MAVS (E) or PKR KO cells (F) were analyzed for the presence of the common EV-associated proteins CD9 and flotillin-1. In addition, the extracellular release of LC3 was analyzed. Representative images of n≥2 independent experiments are shown. * p<0.05, ** p<0.005, *** p<0.0005, **** p<0.0001 based on a two-way one-sample t-test or two-sided t-test.

All tested viral security proteins tested share the ability to suppress SG formation and IFN-β induction. Therefore, we next investigated whether inhibition one or both of these processes plays a role in potentiating EV-enclosed virus release in infected cells. Induction of IFN-β expression in response to EMCV infection requires detection of the virus by the antiviral sensor MDA-5, which subsequently activates the downstream adaptor protein MAVS [30]. In contrast, the formation of stress granules in response to EMCV infection relies on the sensing of the virus by the antiviral sensor PKR [30]. We therefore decided to use KO cell lines for the host factors MAVS and PKR to assess whether preventing the induction of these two antiviral signaling pathways during EMCV-L^Zn^ infection affects EV and EV-enclosed virus release. WT, MAVS KO, and PKR KO cells were infected with EMCV-L^Zn^ or EMCV-Wt as control, followed by comparison of intracellular and EV-enclosed virus titers. Virus titers in MAVS KO cells resembled those observed in WT cells (Fig 3A). Knock out of PKR, on the other hand, negated the difference in intracellular virus titers between EMCV-Wt and EMCV-L^Zn^ infected cells, and reduced the difference in EV-enclosed virus release (Fig 3A). Upon normalization for the potential differences in intracellular virus production, it was clear that EMCV-L^Zn^ induced less EV-enclosed virus release than EMCV-Wt in all cell lines, but that this defect was significantly reduced in PKR KO cells (Fig 3B). In line with this finding, we observed that PKR KO (partially) rescued the increase in overall EV numbers (Fig 3C) as well as the induction of the FSC-high EV subsets carrying flotillin and LC3 during EMCV-L^Zn^ infection (Figs 3D, 3E and S6A). In contrast, the number and composition of EVs released by MAVS KO cells remained comparable to that observed in WT cells upon infection with either virus (Figs 3D, 3F and S6B). These data indicate that the inhibition of the PKR antiviral signaling and downstream SG formation, but not MDA5-MAVS signaling, potentiates the release of EVs and EV-enclosed virus during EMCV infection.

**Fig 4:**
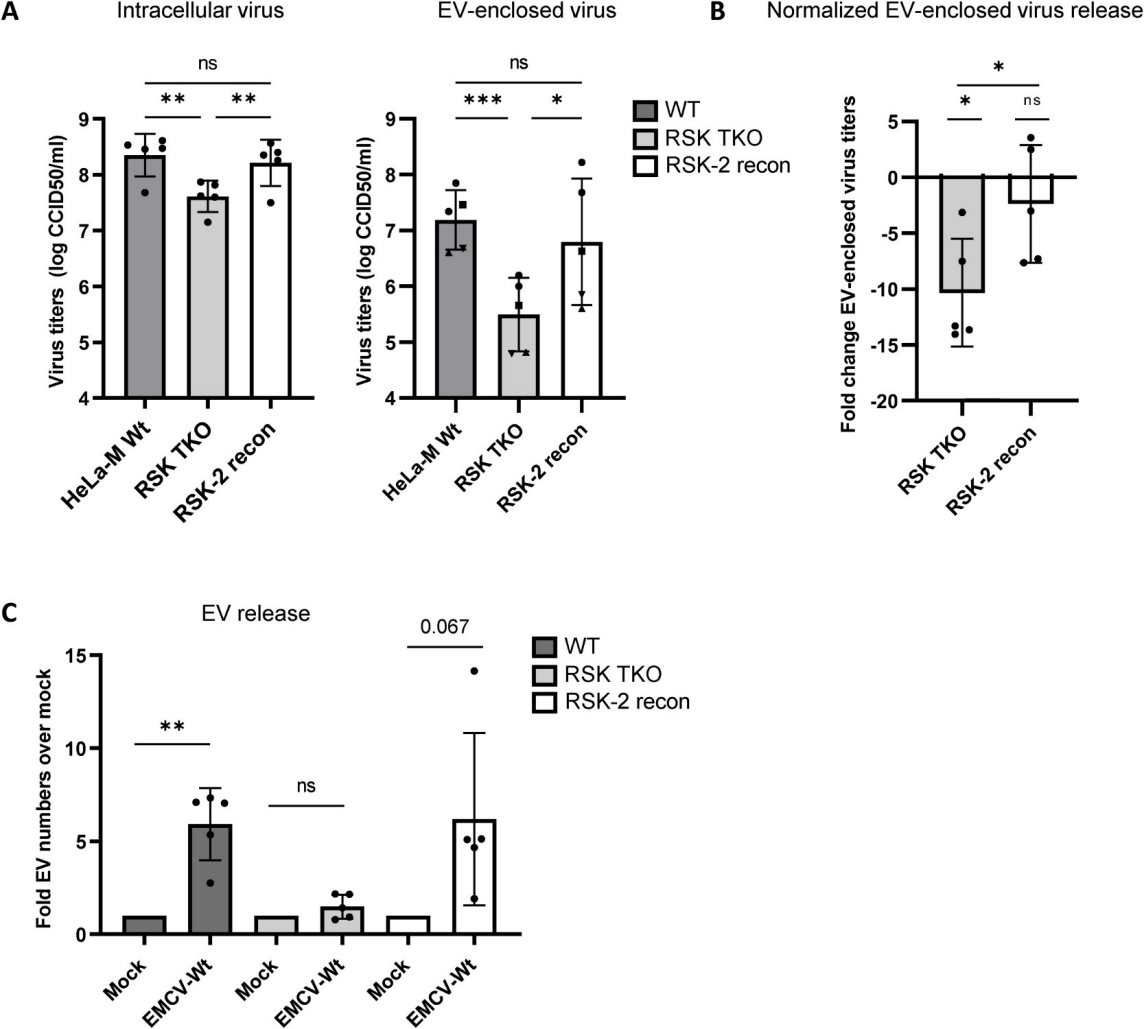
The EMCV Leader requires the host kinase RSK to promote EV release during infection. Hela-M WT cells, RSK-1/2/3 triple KO (TKO) cells, and TKO cells reconstituted with RSK-2 were mock infected or infected with EMCV-Wt. 7 hpi EVs were isolated from the supernatant of (mock) infected cells. (A) Intracellular virus titers and EV-enclosed virus titers were determined by end-point dilution assay. *** p<0.0005, * p<0.05 as determined by a RM one-way ANOVA with Geisser-Greenhouse correction. (B) Depicted is the fold change in EV-enclosed virus titers upon RSK TKO and RSK-2 reconstitution after correcting for the fold change in intracellular virus titers in the corresponding samples. * p<0.05 as determined by a two-tailed t-test or one-sample t-test. (C) EV numbers were quantified by high resolution flow cytometry. Depicted is the fold increase in EV numbers relative to the respective mock-infected controls. ** p<0.005 as determined by a two-tailed one-sample t-test. For (A-C) means ± SD of n = 5 independent experiments are shown.

Inhibition of the PKR antiviral pathway by CVB3 2A^pro^ is believed to rely on the cleavage of (unknown) host factors downstream of PKR. Yet the cardiovirus Leaders were recently described to inhibit PKR activation via physical interaction with and activation of the host kinase RSK [31]. Therefore, we assessed if the knocking out RSK would reduce the ability of EMCV-Wt to induce EV-enclosed virus release. Knock out of RSK-1-3 homologues (TKO) reduced intracellular virus titers (Fig 4A) but caused an even greater decrease in EV-enclosed virus release 7 hpi (Fig 4A, normalized data in Fig 4B). This effect could be restored by reconstitution of RSK-2 (Fig 4A and 4B). In line with these findings, high resolution flow cytometry analysis confirmed that RSK depletion abolished the increase in EV release induced by EMCV infection and that this was (partially) restored upon re-introduction of RSK-2 (Fig 4C). These data further support the notion that inhibition of the PKR antiviral pathway is required for enhanced release of EVs and EV-enclosed viruses during EMCV infection, and that for cardiovirus Leaders this relies on the interaction with the host kinase RSK.

### A role for P38 MAPK in the induction of EV and EV-enclosed virus release by EMCV L and CVB3 2A^pro^

The above data demonstrate an important role for inhibition of the PKR pathway in enabling the production of virus-carrying EV subsets. Yet, recombinant viruses expressing TMEV L or SAFV L were not able to rescue the induction of virus-induced EV subsets (Fig 2A, 2D and 2E), despite causing a similar degree of PKR inhibition as EMCV-Wt (S1A Fig). Furthermore, PKR KO did not fully restore the production of virus-carrying EVs by leader-deficient *vs*. Wt EMCV despite highly similar intracellular virus titers (Fig 3A and 3B). This suggests that viral proteins such as EMCV L modulate additional host cell pathways involved in the regulation of EV-enclosed virus release. Hence, we aimed to identify host cell pathways that were differentially regulated by the recombinant viruses EMCV-L^TMEV/SAFV^ *versus* EMCV-2A^pro^/EMCV-Wt that could explain the observed difference in efficiency by which these two sets of viruses modulate EV-enclosed virus release and induce secretory autophagy.

**Fig 5:**
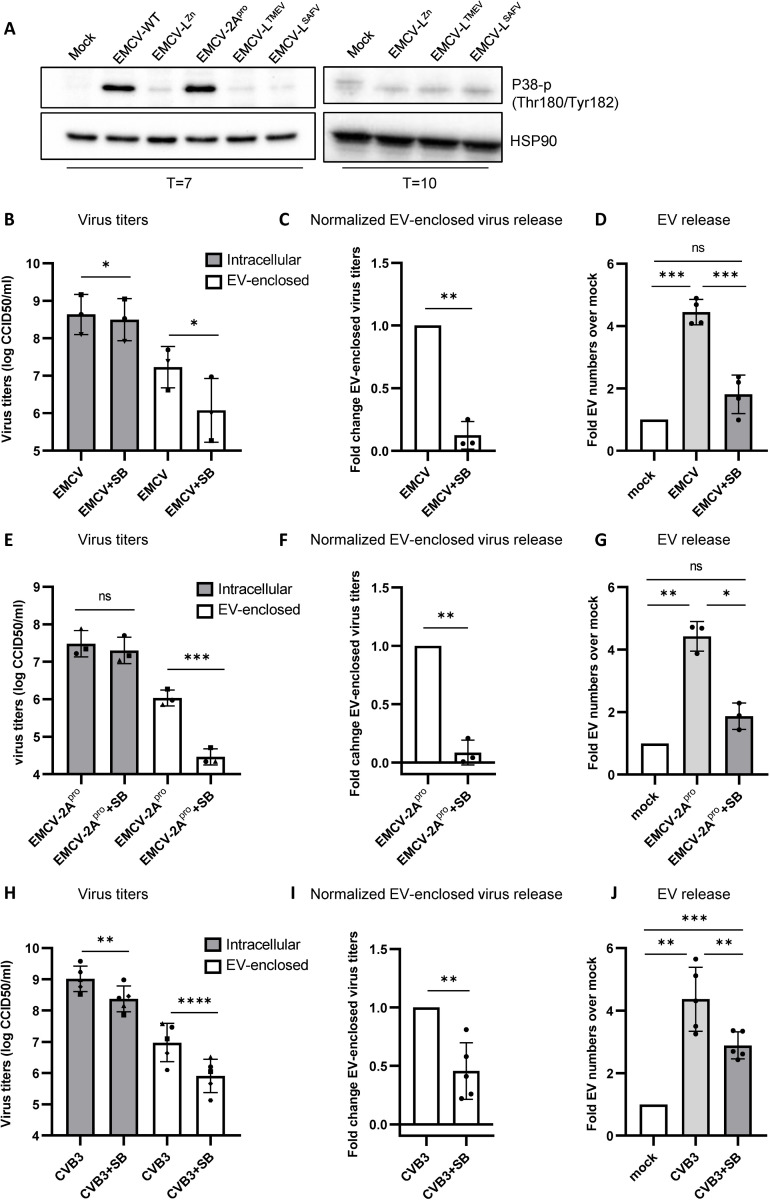
Activation of the stress kinase P38 MAPK in the presence of EMCV L and CVB3 2A^pro^, but not TMEV L and SAFV L, promotes EV and EV-enclosed virus release during infection. (A) Phosphorylated P38 MAPK (Thr 180/Tyr 182) was detected by western blot in whole cell lysates of mock-infected Hela cells or HeLa cells infected with the indicated (recombinant) viruses at 7 and 10 hpi. HSP90 was detected as loading control. Representative images of n = 3 independent experiments are shown. (B-G) EMCV-Wt (B-D) or EMCV-2A^pro^ (E-G) infected cells were treated with 10 μM of the P38 inhibitor SB203580 (SB) 1 hpi. (B,E) Intracellular and EV-enclosed virus titers 7 hpi were determined using end-point dilution assay. (C,F) Quantification of the fold change in EV-enclosed virus titers upon treatment with the P38 inhibitor SB, after correction for differences in intracellular virus production in the corresponding samples. (D, G) EV numbers were quantified by high resolution flow cytometry. Depicted is the fold increase in EV numbers during infection relative to the respective mock-infected controls. (H-J) Experiments indicated above were repeated for CVB3-Wt infected cells treated with 50 μM SB starting 30 min pre-infection. * p<0.05, ** p<0.005, ***p<0.0005, ****p<0.0001 as determined by a paired two-tailed t-test or one-sample t-test. Bar graphs display mean ±SD of n = 3–5 independent samples.

EMCV L was previously shown to promote the activation of the stress kinase P38 MAPK [34]. Notably, P38 has been described as an important regulator of EV release in response to various pro-inflammatory stimuli [45–50]. P38 activation has also been demonstrated in response to CVB3 infection, and was shown to promote CVB3 virus release [51,52]. This prompted us to investigate a potential link between P38 activation by the picornavirus security proteins and the induction of EV-enclosed virus release. In EMCV-Wt and EMCV-2A^pro^ infected cells we observed increased levels of phosphorylated (activated) P38, whereas P38 activation was not observed for EMCV-L^Zn/TMEV/SAFV^ infected cells at 7 or 10 hpi (Fig 5A). To assess whether the observed activation of P38 in EMCV-Wt and EMCV-2A^pro^ infected cells contributed to enhanced induction of EV and EV-enclosed virus release, cells were treated with a low dose of P38 inhibitor SB203580 (SB). The inhibitor had a minimal effect on EMCV-Wt intracellular virus titers, but lead to a clear reduction in EV-enclosed virus release (Fig 5B and 5C). In addition, we observed that P38 inhibition largely abolished the induction of overall EV release in response to infection (Fig 5D). P38 inhibition also strongly inhibited the release of EVs and EV-enclosed viruses in response to infection with EMCV-2A^pro^, whereas no reduction of intracellular virus titers was observed (Fig 5E–5F). In support of these data, we confirmed that inhibition of P38 in cells infected with wild type CVB3 also led to reduced release of EVs and EV-enclosed virus, albeit with a smaller effect size (Fig 5H–5J). Hence, our data highlights a differential capacity of picornavirus security proteins to promote the activation of the P38 stress pathway, and implicates this pathway as a positive regulator for the production of virus-carrying EVs during infection.

Collectively, these data provide new insights into the determinants of EV-enclosed virus release. We show that both the inhibition of the PKR antiviral stress pathway and the activation of the stress kinase P38 pathway regulate the production of EVs and EV-enclosed virions during EMCV infection. Picornavirus security proteins that modulate both pathways are most efficient in inducing the release of virus-carrying EV subsets.

## Discussion

In this study we made use of a recombinant virus expression system to allow for the direct side-by-side comparison of the impact of different picornavirus security proteins on EV and EV-enclosed virus release. Using this approach, we show that functionally related non-structural proteins encoded by viruses belonging to different genera in the family *Picornaviridae* can promote the release of EV-enclosed virus. The Leader proteins of three different members of the genus *Cardiovirus* (i.e. EMCV, TMEV and SAFV) as well as the 2A^pro^ protein of CVB3, a member of the genus *Enterovirus*, all promoted virus packaging within EVs, albeit with different efficiencies. Of these proteins, EMCV L and CVB3 2A^pro^ induced a more rapid and prominent increase in EV-enclosed virus release, correlating with an increase in overall EV production and the induction of flotillin+, LC3+, FSC-high EV subsets. These findings highlight differences in the capacity of these viral proteins to induce EV-enclosed virus release, providing an opportunity to explore the cellular pathways involved in this process.

Using knock out cells we were able to show that suppression of MAVS-mediated IFN induction, a prominent antiviral pathway inhibited by picornaviral security proteins, does not contribute to the induction of EV release during EMCV infection. Previous studies implied an inhibitory effect of IFN-stimulated genes on EV release in the context of infection with the picornavirus EV-A71 [10]. However, the short time window of EMCV replication, in combination with the fact that a single cycle infection was studied, may explain why we did not observe an impact of IFN induction on EMCV-induced EV release.

In contrast to MAVS, deletion of PKR, the antiviral sensor responsible for the formation of stress granules, partially restored the inability of EMCV lacking expression of its security protein EMCV L to induce EV and EV-enclosed virus release. In line with this finding, we observed that the induction of virus-induced EV subsets by EMCV L required the host factor RSK, a host kinase that was recently implicated in the inhibition of PKR by cardioviruses [31,53]. Together, these data suggest that PKR signaling acts as a negative regulator of EV-enclosed virus during infection. Interestingly, knock down of PKR has previously also been described to potentiate EV release in response to the expression of prion proteins [54]. Yet, the mechanism by which PKR signaling affects EV release remains unknown. Whereas cardiovirus Leader proteins mediate partial inhibition of PKR activation itself, CVB3 2A^pro^ inhibits stress granule formation downstream of PKR through mechanisms that remain incompletely understood [38]. However, it remains to be investigated whether stress granule formation is the sole pathway explaining the inhibitory effect of PKR signaling on EV release. Importantly, we observed that TMEV L and SAFV L had a significantly lower capacity to promote EV and EV-enclosed virus release than EMCV L, despite comparable efficiencies in inhibiting PKR signaling and stress granule formation. Combined, the data indicate that virus-induced EV release in our system requires inhibition of PKR activation and is potentiated by PKR KO. Yet, the (partial) inhibition of PKR mediated by viral proteins such as TMEV L and SAFV L is not sufficient to prompt the production of virus-carrying EV subsets seen in the presence of EMCV L/CVB3 2A^pro^.

We show that CVB3 2A^pro^ and EMCV L, but not TMEV/SAFV L, share the ability to promote P38 activation during EMCV infection, and that this is required for induction of EV production and enhanced EV-enclosed virus release. P38 is a central hub for cellular stress pathways and can be activated by a wide variety of stimuli [55]. Transfection of cells with EMCV L was previously shown to be sufficient to induce P38 phosphorylation [34]. This was independent of upstream MAPKKK/MAPKK kinases [34], and may be caused by inhibition of protein phosphatases through EMCV L protein-binding domains. In line with our observation that the cardiovirus Leaders differ in their ability to activate P38, EMCV L was previously shown to employ both P38 and the ERK-RSK pathways to modulate host cell behavior, whereas TMEV L was found to rely predominantly on RSK [31,34,53]. P38 activation has also frequently been described during CVB3 infection [51,52], however the mechanism behind this activation remains elusive. Our data points to a (supporting) role for CVB3 2A^pro^ in this process. For both EMCV and CVB3 WT viruses we confirmed that inhibition of P38 led to a reduction of virus-induced EV release. While the main function previously attributed to the activation of P38 by EMCV L is supporting the induction of NCTD [34], CVB3 2A^pro^ is capable of inducing NCTD through direct cleavage of the nucleopore complex protein Nup98 [40]. Therefore, the observed importance of P38 for EV and EV-enclosed virus release in both EMCV-Wt, EMCV-2A^pro^, and CVB3 infected cells is unlikely to be explained through interference in virus-induced NCTD. Our data indicate that increased release of EV-enclosed viruses is rapidly followed by a loss of cell integrity. The induction of EV-enclosed virus release may therefore be part of a strong cellular stress response, regulated by both P38 and PKR stress pathways, which precedes infection-induced cell death.

In line with our findings in virus-infected cells, P38 has been implicated as a regulator of EV induction in response to various other pro-inflammatory stimuli, including extracellular ATP (eATP), TNF-α, high glucose, and triggering of the platelet-activating factor receptor [45–50]. Release of EVs in response to many of these stimuli was shown to be mediated by acid sphingomyelinase (A-SMase), a lysosomal sphingomyelinase that upon secretion promotes plasma membrane fluidity and subsequent budding to form EVs [47,48,50]. For eATP stimulation, P38 activation was required to increase overall cellular A-SMase activity [50]. In addition, P38 may be involved in the delivery of A-SMase to the plasma membrane, as P38 activity was implicated in relocalization of lysosomes to the cell periphery [56]. Together, these data prompt further investigation into the role of A-SMase in the release of EVs during EMCV and CVB3 virus infection.

In previous work we showed that induction of secretory autophagy contributes to the packaging of virus in EVs during EMCV and CVB3 infection [22,25]. During EMCV infection, the induction of secretory autophagy coincides with a decrease in the efficiency of autolysosome formation [25]. Recruitment of P38 to autophagosomes has been shown to inhibit autophagosome-lysosome fusion via ATG5 phosphorylation [57]. Hence, the observed P38 activation promoted by EMCV L and CVB3 2A^pro^, but not TMEV/SAFV L, potentially contributes to the observed induction of secretory autophagy. Currently, little is known about the regulation of the secretory autophagy pathway. Known triggers for the release of proteins via secretory autophagy include ER stress and proinflammatory signaling, among which inflammasome activation [58–62]. We propose that by affecting cellular stress responses, viral proteins such as EMCV L and CVB3 2A^pro^ contribute to a cell stress phenotype that may promote the activation of such proinflammatory signaling cascades.

An important observation of this study is that the modulation of cellular stress pathways by viral proteins can lead to the activation of specific EV formation pathways in response to infection, and the packaging of virus particles in defined EV subsets. This is important because the type of EV that encloses a virion may influence the functional properties of the particle. Within an EV, a virion is co-packaged with host-derived molecules, which may influence how EV-enclosed virions contribute to the infection. Motifs within the EV membrane, such as integrins and exposed PS lipids, can vary between EV subsets and have been described to affect the biodistribution of EVs *in vivo* as well as the preferential binding to and uptake of EVs in different cell types (reviewed in [63,64]). In addition, different EV subsets can contain different host-derived signaling molecules that are co-incorporated with the virions. These molecules may trigger proinflammatory responses that could limit infection, such as mitochondrial DNA or other DAMPS [65,66], but can also possess proviral functions. For example, enterovirus A71 infected cells were shown to release EVs containing miR-146a, which increased the infection efficiency of virus particles enclosed in the same EV isolates [10]. Our data provides initial evidence that different virus-carrying EVs may be released depending on the type of molecular virus-host interactions occurring inside a cell, prompting further investigations into the functional heterogeneity among EV-enclosed virus particles.

Overall, the findings of this study shed new light on host and virus-encoded regulators of EV-enclosed virus release. We show that multiple picornavirus security proteins share the capacity to promote EV-enclosed virus release during infection. Importantly, the host proteins PKR and P38 MAPK, identified here as modulators of EV release during infection, are known to be widely targeted by a broad range of viruses. In addition to the *picornaviridae*, SARS-CoV-2, influenza A virus, herpes simplex 1, hepatitis C virus, HIV, rotavirus, and many more viruses have been shown to activate P38 to promote infection [67,68]. Similarly, viral factors with the ability to prevent PKR antiviral signaling are widespread throughout the viral kingdom [69]. In addition to known effects of P38 and PKR on virus replication and/or assembly, our work points to an impact of these host kinases on EV-mediated spreading of viruses, which further emphasizes their potential as therapeutic targets.

## Materials and methods

### Cells and viruses

Human cervical carcinoma cells (HeLa R19) and baby hamster kidney cells (BHK21) were purchased from the American Type Culture Collection (Rockville, MD), and buffalo green monkey cells (BGM) from the European Cell Culture Collection (ECACC). The production of PKR and MAVS KO HeLa R19 cells by CRISPR-Cas9 gene editing has been described elsewhere [70,71]. HeLa M WT, RSK-1/2/3 triple KO, and RSK-2 reconstituted cells were a kind gift from dr. prof. T. Michiels (de Duve Institute, Université Catholique de Louvain, Belgium) and have been described in [53]. All cell lines were cultured in Dulbecco’s Modified Eagle Medium (high glucose, GlutaMAX, ThermoFischer Scientific, Waltham, MA, USA), supplemented with 10% fetal calf serum (FCS; GE Healthcare Bio-Sciences, Chicago, IL), 100 U/mL penicillin and 100 μg/mL streptomycin (Gibco, Paisley, United Kingdom) and kept in a humidified incubator at 37°C and 5% CO_2_.

EMCV-Wt and recombinant viruses were produced using the previously described M16.1 infectious cDNA clone of mengovirus containing a shortened poly-C tract [72]. In this clone, C19A, C22A point mutations were introduced to inactivate the EMCV Leader protein (EMCV-L^Zn^) [27]. Recombinant EMCV viruses were generated as described previously [70]. In brief, the coding sequences for TMEV L, SAFV L, and CVB3 2A^pro^ were amplified from infectious cDNA clones of CVB3 Nancy, TMEV DA1, and SAFV3 NL2007 [73–75]. PCR primers were flanked by XhoI/NotI restriction sites and the genes inserted into the corresponding restriction sites of the pStrep2-VFETQG-C19A-C22A-M16.1 infectious clone. In this clone, genes of interest are fused to the polyprotein of EMCV-L^Zn^ separated by a strep2 tag and a 3C cleavage site to liberate the protein of interest from the EMCV polyprotein after production. For CVB3 2A^pro^ the strep2 tag was omitted. A C109A mutation was introduced in CVB3 2A^pro^ by site-directed mutagenesis to generate the catalytically inactive version of CVB3 2A (2A^mut^) [38]. For EMCV virus stock production, *in vitro* transcribed run-off RNA transcripts were transfected into BHK-21 cells. For CVB3 WT virus stock production, run-off transcripts of the p53CB3/T7 infectious clone containing CVB3 strain Nancy were transfected in BGM cells [74]. Upon the observation of complete cytopathogenic effect (CPE), samples were subjected to three consecutive freeze-thaw cycles. Cell debris was removed by centrifugation for 30 min at 3000x*g* and virus purified by pelleting through a 30% sucrose cushion at 25,000 rpm for 15 hrs in a SW32 rotor (k-factor 321) (Beckman Coulter, Brea, CA).

### Antibodies

The following antibodies were used for western blotting: mouse-α-CD9 (1:2000, clone HI9a; Biolegend, San Diego, CA), mouse-α-Flotillin-1 (1:1000, clone 18/Flotillin-1; BD Biosciences), mouse-α-LC3 (1:500, clone 5F10, Nanotools, Teningen, Germany), rabbit-α-LC3 (1:1000, polyclonal; MBL international, Woburn, MA), rabbit-α-phospho P38 MAPK (1:2000, polyclonal, Promega), mouse-α-HSP90 (1:1000, clone 68/HSP90, BD Biosciences), rabbit-α-phospho PKR (1:1000, clone E120; Abcam), mouse-α-actin (1:30,000, clone AC-15, Sigma-Aldrich), mouse-α-GAPDH (1:2000, clone mAbcam 9484; Abcam), HRP-coupled goat-α-mouse secondary antibody (1:10,000; Jackson ImmunoResearch Labaratories Inc., West Grove, PA), goat-α-rabbit secondary antibody (1:2500, P0448, DAKO, Denmark). For immunofluorescence staining cells were stained with rabbit-α-G3BP1 (1:150, polyclonal, Aviva, San Diego, CA), mouse-α-hnRNPK (1:100, clone D-6, Santa Cruz), goat-α-mouse or goat-α-rabbit Alexa488 and 647 (1:200, polyclonal, Invitrogen, MA), donkey-α-mouse Alexa488 and 647 (1:200, polyclonal, Thermo Fischer Scientific).

### EV isolation

Cells were infected at a multiplicity of infection (MOI) of 10. After 1hr incubation, cells were washed three times with PBS +Ca+Mg and incubated with cell culture medium containing 10% EV-depleted FCS. To this generate EV-depleted FCS, FCS was prediluted 1:3 in DMEM, ultracentrifuged for 16–20 hrs at 28,000 rpm in an SW32 rotor (k-factor 256.8) and passed through a 0.22 μm filter. For inhibition of P38 during EMCV-Wt/EMCV-2A^pro^ infection, EV-depleted medium was supplemented with 10 μM SB203580 (Selleckchem, The Netherlands). For CVB3 samples, 50 μM SB203580 was added 30 min prior to infection and maintained in the medium all steps thereafter as described previously [52]. At the indicated time points post infection, cell culture supernatants were harvested and centrifuged 10 min at 200x*g* and 2x 10 min at 500x*g* to remove cells and cell debris. For recombinant virus infection experiments, samples were in addition treated with 100 μg/ml DNase I (Roche) for 45 min at 37°C prior to EV isolation. EVs were isolated from clarified supernatants by UC pelleting for 65 min at 28,000 rpm in an SW40 rotor (k-factor 144.5) or by polyethylene glycol (PEG) precipitation o/n (10% PEG6000, 75mM NaCl, 5mM EDTA, 75mM Tris-HCl). EV pellets were resuspended in PBS or PBS +0.1% BSA (cleared from aggregates by ultracentrifugation for 16–20 hrs at 100,000x*g*). Next, EVs were mixed with iodixanol (Optiprep; Axis-Shield, Oslo, Norway) to a final concentration of 45% and overlaid with a linear gradient of 40%-5% iodixanol in PBS. Density gradients were centrifuged at 192,000x*g* for 16 hrs in a SW40 rotor. Gradient fractions of 1 mL were collected from the top and their densities assessed by refractometry. Individual gradient fractions were analyzed for their EV/virus content and the data of fractions within the density range of EVs (1.10–1.06 g/ml) was pooled for analysis unless otherwise indicated.

### Virus titration

Virus titers in EVs were determined by performing end-point dilution assay. BHK-21 cells or HeLa-R19 cells were infected with 3 to 5-fold serial dilutions of prediluted density gradient fractions, and virus titers expressed in Median Cell Culture Infectious Dose (CCID50) values were calculated 3 days after infection using the Spearman-Karber calculation method for EMCV and CVB3 respectively. For intracellular virus titration, adherent cells and cells pelleted from the cell culture supernatant were combined and freeze-thawed three times. Cell debris was removed by centrifugation for 10 min at 500x*g* prior to virus titration.

### High resolution flow cytometry

For single particle characterization of EV release, EVs were fluorescently labeled with 30 μM CFSE (Invitrogen, Carlsbad, CA) for 1 hr at RT prior to purification by density gradient centrifugation. Individual gradient fractions were mixed vigorously with 2% PFA and fixed for 30 min at RT. Samples were diluted 1:20 in PBS immediately prior to high-resolution flow cytometric analysis on a BD Influx flow cytometer with optimized configuration, as previously reported [43,44]. In short, thresholding was applied on fluorescence generated by CFSE-labeled EVs passing the 488 nm laser. Fluorescent 200 nm polystyrene beads (FluoSpheres, Invitrogen) were used to calibrate the fluorescence, forward (FSC) and side (SSC) scattered light settings. Samples were measured at low pressure (sheath fluid: 5 PSI, sample: 4.2 PSI) using a 140 μm nozzle with event rates below 10,000 per second. Measurements were acquired in a fixed time window of 30 seconds to allow direct comparison of EV concentrations in parallel samples. For Fig 5J, samples were measured on the Cytek Aurora spectral flow cytometer instead, using optimized settings for the detection of small particles. A similar fluorescent thresholding approach was applied, and for each sample a fixed volume was measured to allow for quantitative comparison of EV release. Data analysis was performed using FlowJo v10.07 software (FlowJo LLC, Ashland, OR) or FCS express v3 (De Novo software, Los Angeles, CA).

### Western blot

Trichloroacetic acid (TCA) precipitation was performed on pooled gradient fractions within the 1.10–1.06 g/ml density range to enable protein analysis of density gradient purified EVs. In brief, samples were supplemented with 125 μg/ml sodium deoxycholate and 10% ice-cold TCA and pelleted for 15 min at 15,000x*g*. Pellets were washed twice with ice-cold aceton and resuspended in Laemmli sample buffer (LSB: 62.5 mM Tris-HCl pH 6.8, 2% SDS, 10% glycerol) with or without 20 mM 2-mercaptoethanol. For analysis of cells and UC pellets, samples were lysed in RIPA buffer (40 mM Tris-HCl pH 8, 0.5% sodium deoxycholate, 1% Triton X-100, 150 mM sodium chloride, 0.1% sodium dodecyl sulfate) supplemented with protease and phosphatase inhibitor cocktails (Roche) prior to mixing with LSB. Cell lysates were cleared by centrifugation at 15,000×*g* for 15 min and protein concentration was determined using a Pierce BCA assay kit (Thermo Fischer Scientific, Waltham, MA) according to the manufacturer’s protocol. For purified EVs or UC pellets, equal number of producing cells were used to normalize input. LC3-I and LC3-II positive controls were purchased from Nanotools (Teningen, Germany). Proteins were denatured by incubating 4 min at 98°C, separated on sodium dodecyl sulfate-polyacrylamide gels by electrophoresis (SDS-PAGE), and transferred to Immobilon PVDF membranes (Merck Millipore Ltd., Cork, Ireland) by wet transfer. Membranes were blocked with 0.25% (v/v) fish skin gelatin (FSG; Sigma-Aldrich) or 5% BSA in PBS + 0.1% tween (PBS-T) for 1 hr at RT, followed by incubation o/n with primary antibodies. Membranes were washed 5x in 0.25% (v/v) FSG or 0.5–1% BSA in PBS-T followed by incubation for 45 min at RT with secondary antibodies. After 3x wash in PBS-T and ≥2x wash in PBS, membranes were developed using ECL solution (SuperSignal West Dura Extended Duration Substrate, Thermo Fischer Scientific) for detection on a Bio-Rad ChemiDoc imager. Images were analyzed by Image Lab software (Bio-Rad). For the detection of phosphoproteins, PBS was replaced with Tris-buffered saline in all buffers.

### Cell permeability assay

To assess the proportion of permeable cells, cells were harvested by trypsinization and pooled with any detached cells recovered from the supernatant following centrifugation at 200x*g* for 10 min. Cells were washed twice in ice cold PBS followed by staining on ice for 30 min using 1:1000 diluted Fixable Viability Dye eFluor 506 (eBioscience, San Diego, CA) according to manufacturer’s instructions. Unbound dye was removed by washing with PBS and the cells were fixed in 1–2% PFA. Cells were analyzed using a CytoFLEX LX (Beckman Coulter). Data analysis was performed using FlowJo. Heat shocked cells were taken along in all experiments as positive control. To this end, cells were incubated for 3 min at 65°C followed by immediate placement on ice for 1 min.

### Immunofluorescence staining

Cells were fixed with 4% PFA 6–7 hpi and permeabilized by incubation with 0.1% Triton X-100 for 10 min. Samples were washed and incubated in block buffer (PBS + 2% BSA + 50mM NH_4_Cl) for 30 min prior to staining for 45 min with primary antibodies diluted in block buffer. After three washes with block buffer, antibody labelling was repeated with secondary antibodies plus 300nM DAPI. Finally, coverslips were washed in block buffer followed by MiliQ before being mounted in FluorSafe (Calbiochem, San Diego, CA) or ProLong Diamond Antifade Mountant (Thermo Fischer Scientific). Samples were imaged using a 60x or 100x CFI PLAN APO oil objective on a Nikon A1R confocal microscope with hardware settings to image the relevant channels sequentially using recommended emission spectral filter settings. Images were processed using ImageJ software.

### Quantitative reverse transcription PCR (RT-qPCR)

Total RNA from cells was extracted using NucleoSpin RNA kit (MACHEREY-NAGEL GmbH & Co. KG). Reverse transcription was performed with the RevertAid first-strand cDNA synthesis kit (Thermo Scientific, CA, USA). The levels of IFN-β RNA were detected by RT-qPCR using Fast SYBR Green Master Mix (Thermo Fisher Scientific, USA) and LightCycler 480 real-time PCR detection system (Roche). The thermal cycling conditions were composed of an initial denaturation step at 95°C for 5 min, 45 cycles at 95°C for 10s, 60°C for 5s and 72°C for 30s. The experiments were carried out in duplicate for each data point. The primer sequences used for quantification were as follows: IFN-β: Fw 5’ ATGACCAACAAGTGTCTCCTCC 3’, Rev: 5’ GCTCATGGAAAGAGCTGTAGTG 3’. β-Actin: Fw 5’ CCTTCCTGGGCATGGAGTCCTG 3’, Rev: 5’ GGAGCAATGATCTTGATCTTC 3’.

### Statistical analysis

Data display and analysis was performed using GraphPad Prism version 9 (GraphPad Software, CA).

## Supporting information

S1 Fig TMEV L, SAFV L, and CVB3 2A^pro^ can suppress stress granule formation, induce NCTD, and prevent IFN-β induction during infection with EMCV-L^Zn^. Cells were infected with EMCV-Wt, EMCV-L^Zn^, or recombinant viruses in which the coding sequences of TMEV L (L^TMEV^), SAFV L (L^SAFV^), or CVB3 2A (2A^pro^) were fused to the polyprotein of EMCV-L^Zn^. (A) Detection of phosphorylated PKR in whole cell lysates of infected cells compared to an uninfected control 7 hpi. (B) The formation of stress granules was monitored by staining for the stress granule marker G3BP1 (red) and DAPI (blue) in (mock) infected cells 6–6.5 hpi. For mock and EMCV-L^SAFV^ expressing samples, brightness was increased 20% to allow visualization of the diffuse signal. (C) The induction of NCTD was monitored by analyzing changes in the cellular distribution of the nuclear protein hnRNPK (hot cyan) compared to DAPI (red). Representative images of n = 3 independent experiments are shown. (D) IFN-β mRNA levels were determined by RT-qPCR 6–6.5 hpi. Bar graphs display the mean IFN-β expression levels ± SD relative to those detected in EMCV-L^Zn^ infected samples (set to 100%) of n = 3 independent experiments. **p<0.005, ***p<0.0005 using a two-tailed one sample t-test.
(TIF)

S2 Fig Induction of extracellular virus release during infection with EMCV-Wt and EMCV-2A^pro^. (A-B) Fold increase in extracellular virus titers observed during infection with the viruses EMCV-Wt and EMCV-2A^pro^ compared to EMCV-L^Zn^. (A) Line graphs depicting results without normalization. (B) Line graph depicting results after normalization for differences in intracellular virus production. Plotted are means ±SD of n = 3–4 independent experiments.
(TIF)

S3 Fig Cell permeability upon infection with EMCV recombinant viruses. (A) The percentage of cells maintaining plasma membrane integrity over the course of infection was measured by staining cells with a cell impermeable dye followed by flow cytometric analysis. Line graphs depict mean ± SD of n = 2–3 independent experiments.
(TIFF)

S4 Fig The impact of reconstitution of EMCV-L^Zn^ with proteolytically active or inactive CVB3 2A^pro^ on intracellular virus titers and EV release. Cells were infected with EMCV-L^Zn^, EMCV-L^Zn^ reconstituted with CVB3 2A^pro^ (EMCV-2A^pro^) or with a mutated version of CVB3 2A that lacks proteolytic activity (EMCV-2A^mut^). (A) Intracellular virus titers were compared 7 hpi using end-point dilution assay. (B) EV numbers were quantified by high resolution flow cytometry. Depicted are the EV numbers relative to the respective mock-infected samples. ns, p>0.05, p**<0.005, ***p<0.0001 as determined by one-way ANOVA with Tukey’s multiple comparisons test. Bar graphs display mean ±SD of n = 3 independent experiments.
(TIF)

S5 Fig Analysis of LC3 in gradient purified EVs upon EMCV recombinant virus infection. Depicted are the expanded western blots shown in Fig 2D including the positive controls for LC3I and LC3II, measured using short (top) and long (bottom) exposure times. Depicted are representative images of n = 2 independent experiments.
(TIF)

S6 Fig Knock out of the antiviral sensor PKR, but not MAVS, rescues FSC^high^ EV release during infection with EMCV-L^Zn^. PKR KO (A) and MAVS KO (B) cells were infected with EMCV-Wt or EMCV-L^Zn^ at MOI 10. 7 hpi EVs were isolated by density gradient centrifugation and light scattering patterns induced by EVs were assessed by high resolution flow cytometry. Bar graphs display the percentage of EVs that display a high degree of forward-scattered light (FSC^high^), based on the gating strategy depicted in Fig 3D. Depicted are means ±SD of n = 3/4 independent samples. * p<0.05, ** p<0.005 determined by one-way ANOVA with Tukey’s multiple comparisons test.
(TIF)

S1 Data Source data file containing individual numerical data points for each of the graphs in this manuscript. (XLSX)
