# Supplementary figures and images for: Picornavirus security proteins promote the release of extracellular vesicle enclosed viruses via the modulation of host kinases
Source: PLoS Pathog. 2024 Apr 25;20(4):e1012133. doi: 10.1371/journal.ppat.1012133 (PMC11075854; doi:10.1371/journal.ppat.1012133)

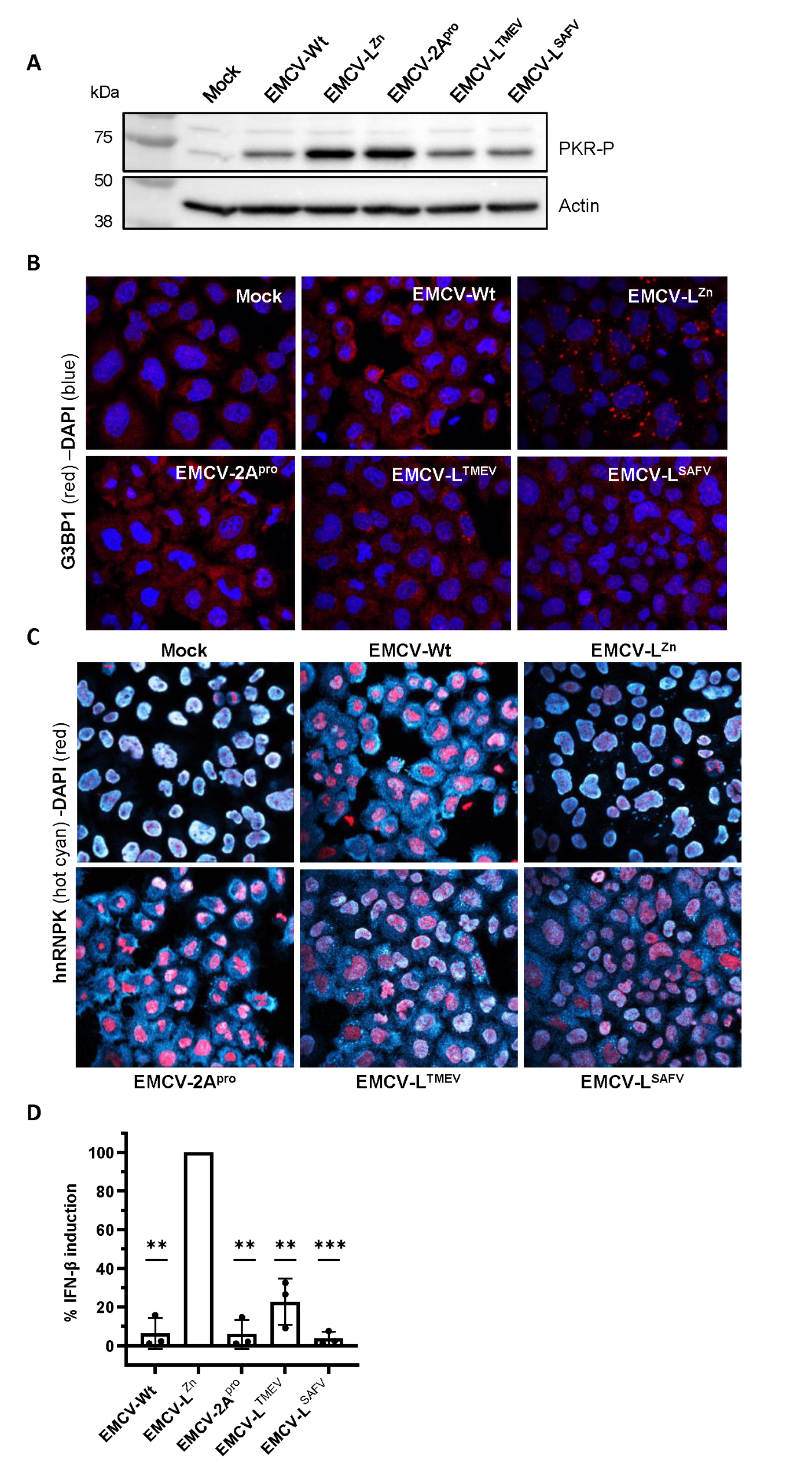

Supplement: S1 Fig — Cells were infected with EMCV-Wt, EMCV-LZn, or recombinant viruses in which the coding sequences of TMEV L (LTMEV), SAFV L (LSAFV), or CVB3 2A (2Apro) were fused to the polyprotein of EMCV-LZn. (A) Detection of phosphorylated PKR in whole cell lysates of infected cells compared to an uninfected control 7 hpi. (B) The formation of stress granules was monitored by staining for the stress granule marker G3BP1 (red) and DAPI (blue) in (mock) infected cells 6–6.5 hpi. For mock and EMCV-LSAFV expressing samples, brightness was increased 20% to allow visualization of the diffuse signal. (C) The induction of NCTD was monitored by analyzing changes in the cellular distribution of the nuclear protein hnRNPK (hot cyan) compared to DAPI (red). Representative images of n = 3 independent experiments are shown. (D) IFN-β mRNA levels were determined by RT-qPCR 6–6.5 hpi. Bar graphs display the mean IFN-β expression levels ± SD relative to those detected in EMCV-LZn infected samples (set to 100%) of n = 3 independent experiments. **p<0.005, ***p<0.0005 using a two-tailed one sample t-test. (TIF) [file ppat.1012133.s001.tif]

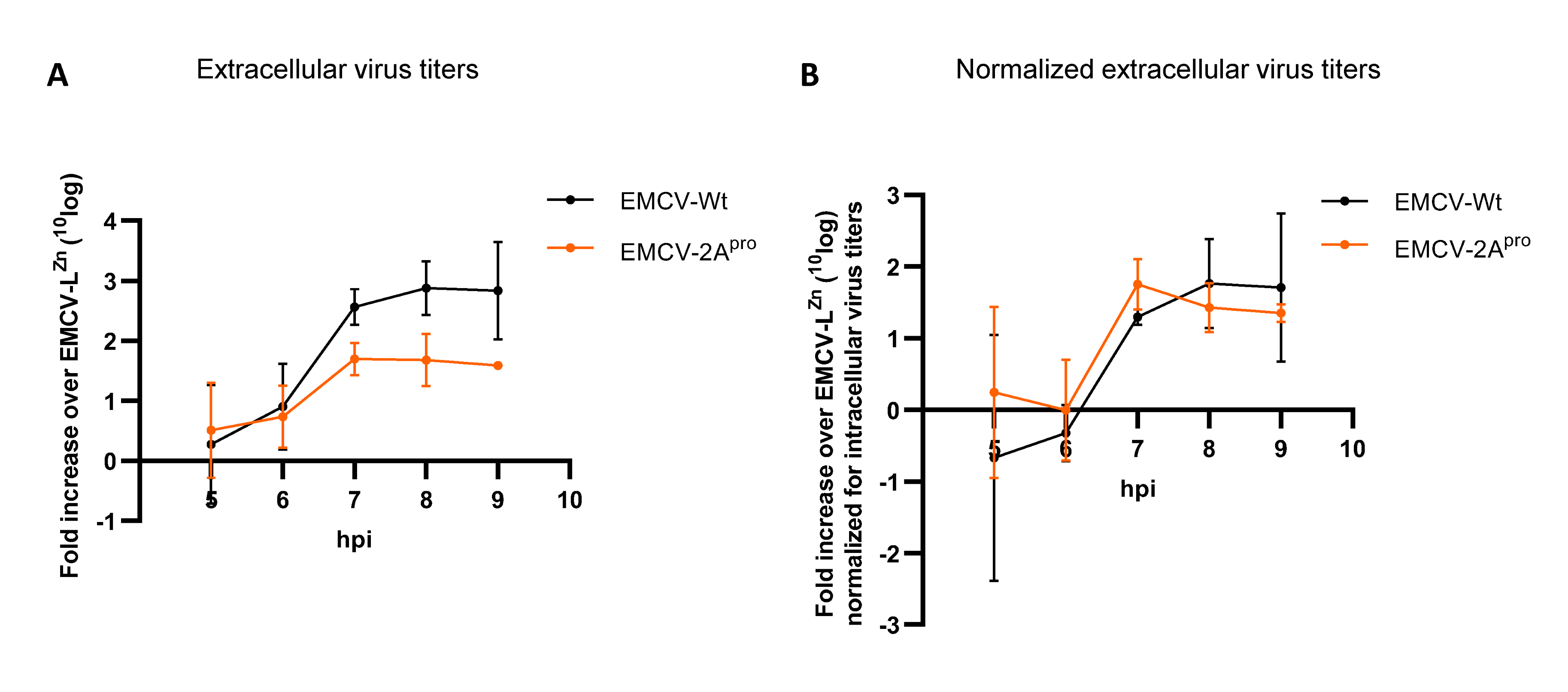

Supplement: S2 Fig — (A-B) Fold increase in extracellular virus titers observed during infection with the viruses EMCV-Wt and EMCV-2Apro compared to EMCV-LZn. (A) Line graphs depicting results without normalization. (B) Line graph depicting results after normalization for differences in intracellular virus production. Plotted are means ±SD of n = 3–4 independent experiments. (TIF) [file ppat.1012133.s002.tif]

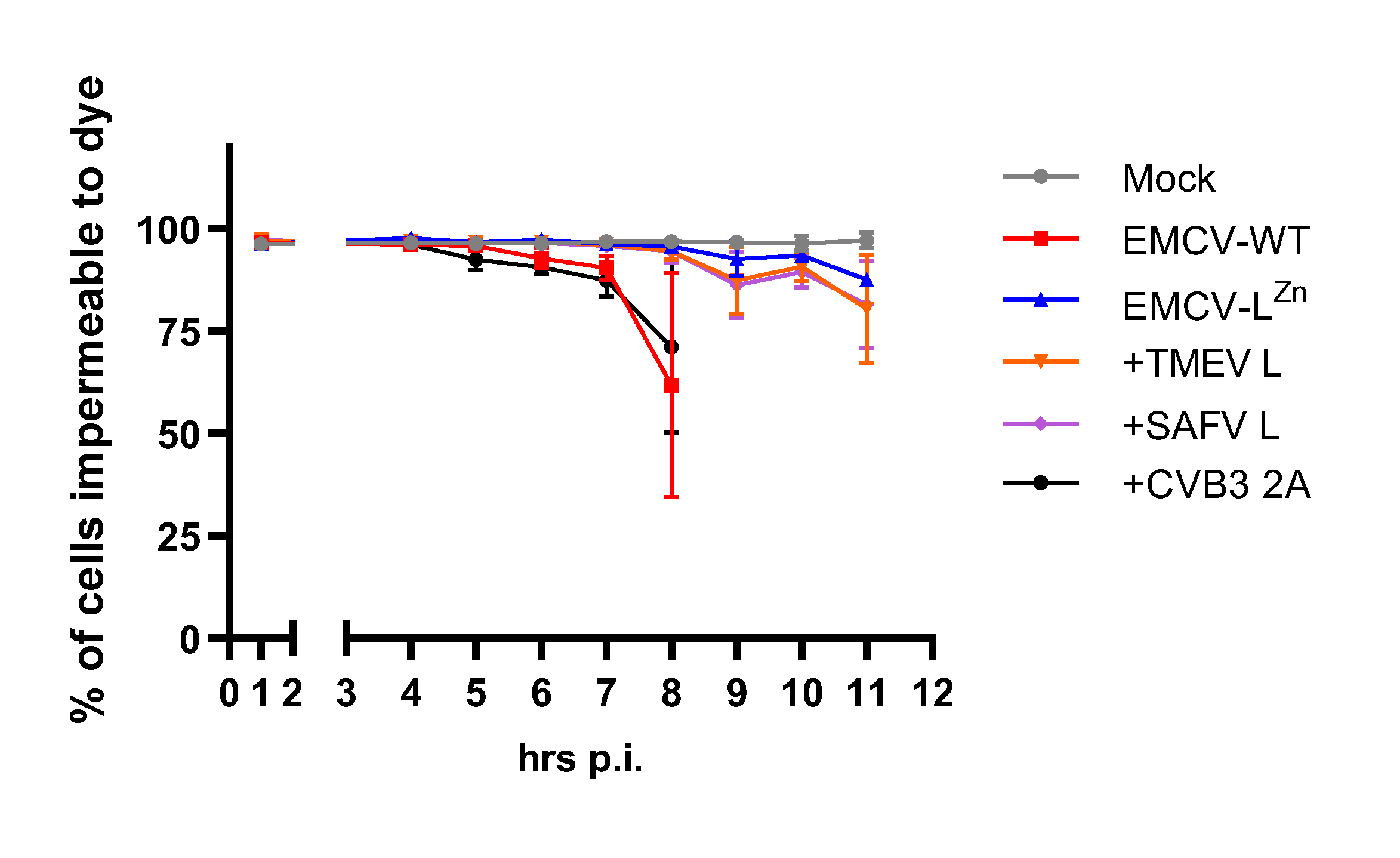

Supplement: S3 Fig — (A) The percentage of cells maintaining plasma membrane integrity over the course of infection was measured by staining cells with a cell impermeable dye followed by flow cytometric analysis. Line graphs depict mean ± SD of n = 2–3 independent experiments. (TIFF) [file ppat.1012133.s003.tiff]

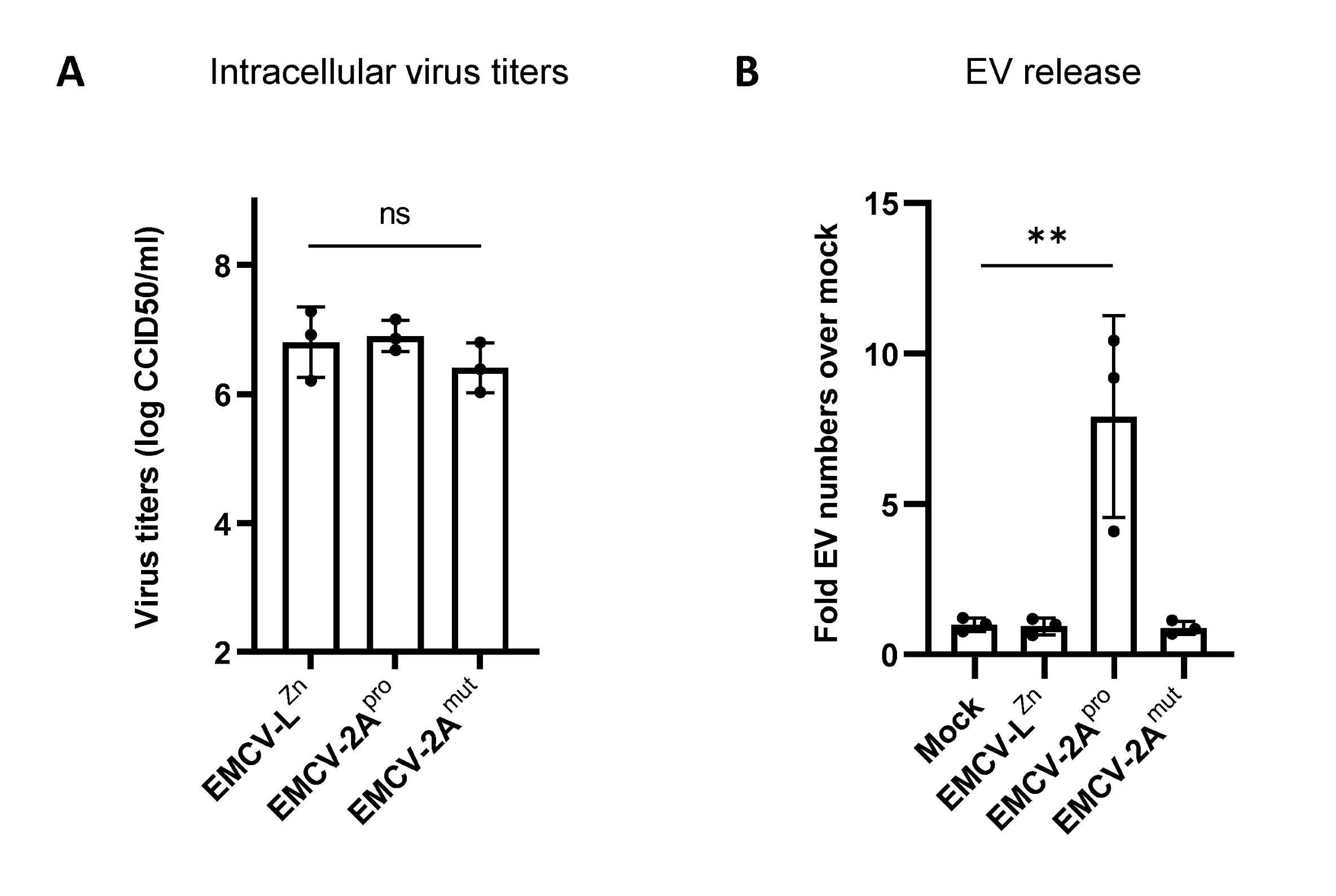

Supplement: S4 Fig — Cells were infected with EMCV-LZn, EMCV-LZn reconstituted with CVB3 2Apro (EMCV-2Apro) or with a mutated version of CVB3 2A that lacks proteolytic activity (EMCV-2Amut). (A) Intracellular virus titers were compared 7 hpi using end-point dilution assay. (B) EV numbers were quantified by high resolution flow cytometry. Depicted are the EV numbers relative to the respective mock-infected samples. ns, p>0.05, p**<0.005, ***p<0.0001 as determined by one-way ANOVA with Tukey’s multiple comparisons test. Bar graphs display mean ±SD of n = 3 independent experiments. (TIF) [file ppat.1012133.s004.tif]

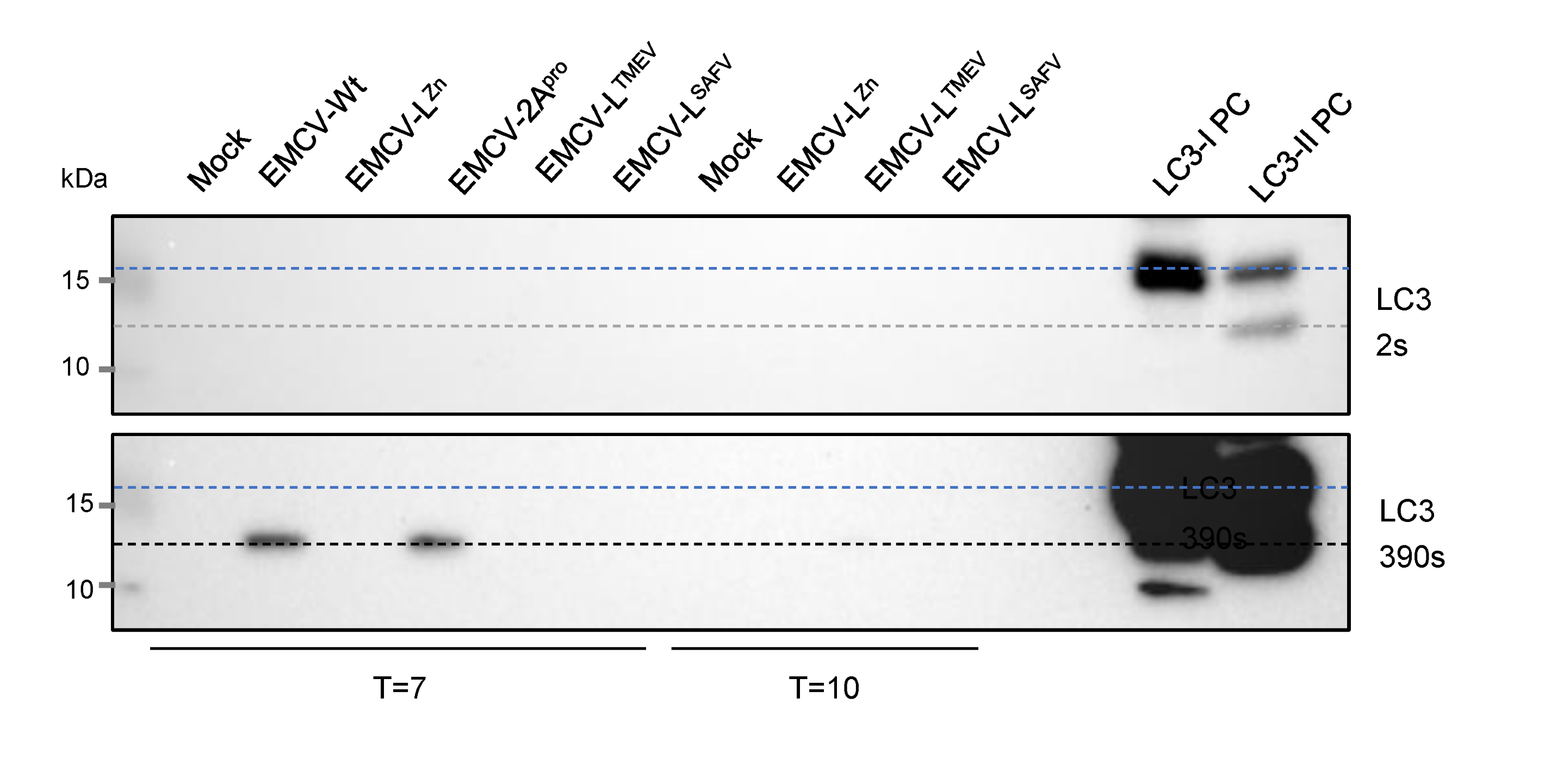

Supplement: S5 Fig — Depicted are the expanded western blots shown in Fig 2D including the positive controls for LC3I and LC3II, measured using short (top) and long (bottom) exposure times. Depicted are representative images of n = 2 independent experiments. (TIF) [file ppat.1012133.s005.tif]

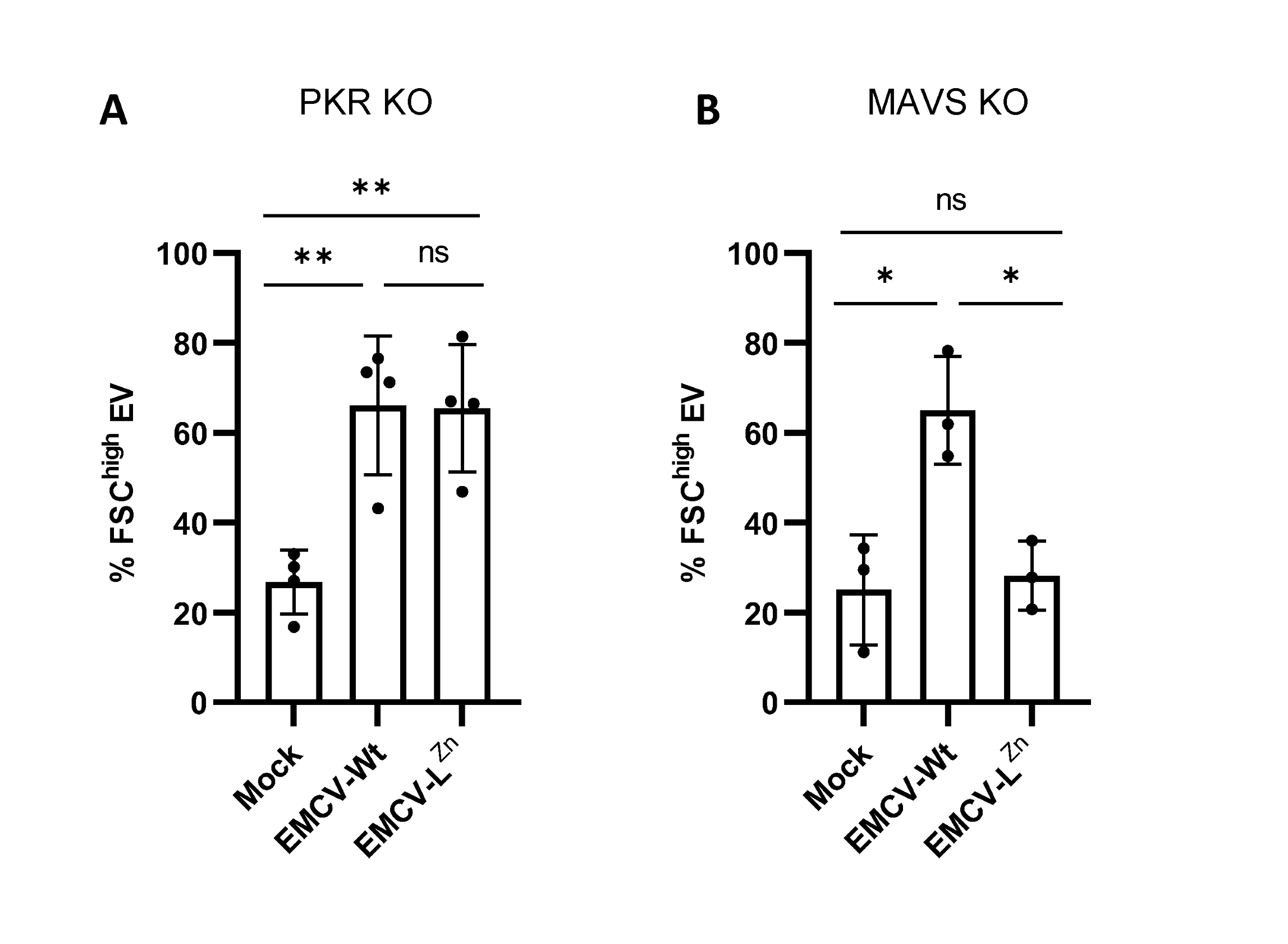

Supplement: S6 Fig — PKR KO (A) and MAVS KO (B) cells were infected with EMCV-Wt or EMCV-LZn at MOI 10. 7 hpi EVs were isolated by density gradient centrifugation and light scattering patterns induced by EVs were assessed by high resolution flow cytometry. Bar graphs display the percentage of EVs that display a high degree of forward-scattered light (FSChigh), based on the gating strategy depicted in Fig 3D. Depicted are means ±SD of n = 3/4 independent samples. * p<0.05, ** p<0.005 determined by one-way ANOVA with Tukey’s multiple comparisons test. (TIF) [file ppat.1012133.s006.tif]
